# *TRiP*: a transfer learning based rice disease phenotype recognition platform using SENet and microservices

**DOI:** 10.3389/fpls.2023.1255015

**Published:** 2024-01-24

**Authors:** Peisen Yuan, Ye Xia, Yongchao Tian, Huanliang Xu

**Affiliations:** ^1^ College of Artificial Intelligence, Nanjing Agricultural University, Nanjing, China; ^2^ College of Agriculture, Nanjing Agricultural University, Nanjing, China

**Keywords:** rice disease recognition, SENet, transfer learning, machine learning as service, microservices framework

## Abstract

Classification of rice disease is one significant research topics in rice phenotyping. Recognition of rice diseases such as *Bacterialblight*, *Blast*, *Brownspot*, *Leaf smut*, and *Tungro* are a critical research field in rice phenotyping. However, accurately identifying these diseases is a challenging issue due to their high phenotypic similarity. To address this challenge, we propose a rice disease phenotype identification framework which utilizing the transfer learning and SENet with attention mechanism on the cloud platform. The pre-trained parameters are transferred to the SENet network for parameters optimization. To capture distinctive features of rice diseases, the attention mechanism is applied for feature extracting. Experiment test and comparative analysis are conducted on the real rice disease datasets. The experimental results show that the accuracy of our method reaches 0.9573. Furthermore, we implemented a rice disease phenotype recognition platform based microservices architecture and deployed it on the cloud, which can provide rice disease phenotype recognition task as a service for easy usage.

## 1 Introduction

Plant phenotype (Zhou et al., 2023) represents the visible morphological characteristics of plants within a specific environment, which plays significant role in areas such as plant protection, breeding, and so on (Kolhar and Jagtap, 2023; Pan et al., 2023). Rice is one of main global crops, and has gained a great deal of attention in plant science, especially regarding phenotypic identification research. These investigations are crucial for generating socio-economic benefits (Xu et al., 2020a). However, the increasing prevalence of rice diseases, exacerbated by fluctuating agricultural practices and climate change, has compromised yield, quality, and the economic viability of rice cultivation (Shahriar et al., 2020; Klaram et al., 2022).

This paper concentrates on the following rice diseases and their phenotypic characteristics: *Bacterial blight*, caused by *Xanthomonas oryzae*, leads to leaf yellowing and wilting, significantly reducing yield. *Blast*, induced by *Magnaporthe oryzae*, ranks among the most devastating global rice diseases, causing grain sterility and yield reduction. *Brown spot*, attributable to *Bipolaris oryzae*, manifests as brown leaf spots, adversely affecting grain quality and yield. *Leaf smut*, resulting from *Entyloma oryzae*, produces black, powdery spores on leaves, severely impairing photosynthesis. *Tungro*, a viral affliction, leads to stunted growth and leaf yellowing, causing substantial damage in affected regions, though it is less widespread.

Rice disease phenotype recognition is an important research field in phytoprotection, which includes traditional manual recognition, classical machine learning-based image processing, and deep learning-based techniques (Hasan et al., 2019). Traditional approaches rely heavily on the visual expertise of trained professionals, demanding extensive experience and knowledge (Patil and Burkpalli, 2021). The advent of smart phytoprotection leverages machine learning capabilities attracted a lot of attention and gained huge success. However, traditional image processing methods often involve manual feature extraction, a process that proves inefficient for disease image feature extraction.

The application of deep learning in phenotype recognition (Xiong et al., 2021) automates feature extraction and inference, which has become a preferred method for identifying rice diseases. With the emergence of various network models such as VGG16 (Chen et al., 2020), Xception (Nayak et al., 2023; Sudhesh, 2023), and Inception (Yang et al., 2023), optimized for plant disease phenotyping, the accuracy of recognition has seen a significant enhancement compared to traditional methods. For instance, Picon et al. (2019) addressed the issue of yield losses due to fungal infections, proposing the use of deep convolutional neural networks for automatic disease image recognition, thereby improving treatment effectiveness and minimizing yield losses. Xu et al. (2020) developed a two-stage RiceNet, utilizing YoloX for detection and a Siamese network for rapid and precise rice disease identification, enhancing rice yield and quality. K.M. et al (Sudhesh, 2023). introduced an attention-driven preprocessing mechanism based on dynamic modal decomposition for rice leaf disease identification. Wang et al. (2021) proposed an attention-based depthwise separable neural network combined with a Bayesian optimization model for efficient detection and classification of rice diseases from leaf images.

Nevertheless, deep learning models, characterized by their complex structures and extensive parameter sets, necessitate substantial data for effective model training. Transfer learning, involving the adaptation of knowledge from a pre-trained model to a new, related task, has emerged as a method to augment the performance of target tasks in plant disease identification (Hossain et al., 2020; Feng et al., 2021; Feng et al., 2022). Zhao et al. (2022) proposed a transfer learning-based approach for identifying corn leaf diseases in natural scene images.

Currently, Machine Learning as a Service (MLaaS) is emerging as a new trend for deploying machine learning applications (Ribeiro et al., 2015; Li et al., 2017; Wang et al., 2018). Deployed on cloud infrastructure, MLaaS facilitates the task reasoning services (Ribeiro et al., 2015). Integrating MLaaS with a microservices architecture enhances its advantages, offering scalable approach for developing and deploying machine learning applications (Labreche et al., 2022; Lohit et al., 2022). This approach allows developers to integrate machine learning into their application architectures without the need to manage infrastructure or possess specialized skills (Manley et al., 2022).

This paper propose a **T**ransfer learning-based **Ri**ce disease phenotype recognition **P**latform (TRiP for short), which integrates the advanced SENet neural network and a microservices architecture. TRiP leverages the cutting-edge concept of machine learning as a service and offers scalable and adaptable solutions for the dynamic needs of rice disease diagnostics (Cherradi et al., 2017; Yi et al., 2019; Daradkeh and Agarwal, 2023).

The contributions are summarized as follows: (1) proposed and developed an innovative rice disease phenotype recognition framework, combining the power of transfer learning with the SENet architecture; (2) The implementation of a customized attention mechanism within the SENet network significantly enhances its ability to focus on and accurately extract features critical to identifying rice diseases; (3) The microservices architecture of TRiP platform brought advanced computational capabilities to edge computing in for rice disease recognition.

## 2 Material

### 2.1 Rice disease dataset

**Figure 1:**
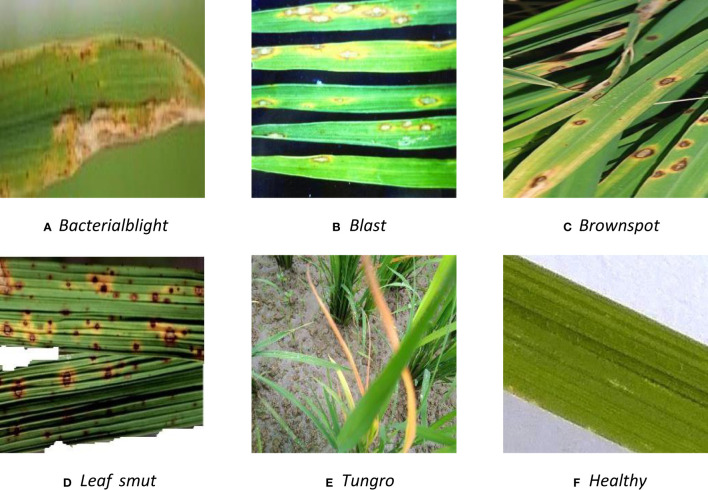
Example of rice disease dataset.

The dataset of rice disease is selected from IP102 and icgroupcas (http://www.icgroupcas.cn/). IP102 is a dataset of crop disease and pest data and widely used in crop disease research. It contains 75,000 images of 102 crop diseases and pests, which covers a wide range of crops including rice, wheat, corn, tomato, etc. In this paper, five diseases *Bacterialblight*, *Blast*, *Brownspot*, *Leaf smut*, *Tungro* are selected from IP102 dataset, because they are the most prevalent rice diseases and can cause significant yield losses. **Figure 1** illustrates the examples of the rice disease images and the healthy one.

In our experiments, each category of the rice pest dataset was randomly divided into the training set and the test set at the rate of 7:3. The training set contained a total of 4322 pictures, the test set contained a total of 1853 pictures, and the whole dataset contained a total of 6175 pictures. **Table 1** shows the detail of the rice disease image dataset used in this paper.

**Table 1:** IP102 rice disease image dataset.

ID	Name	Training set size	Test set size
1	*Bacterialblight*	1111	476
2	*Blast*	1008	432
3	*Brownspot*	1120	480
4	*Leaf smut*	28	12
5	*Tungro*	915	393
6	*Healthy*	140	60

### 2.2 Dataset for transfer learning

The dataset used for transfer learning in this paper was selected from the *PlantVillage* dataset to ensure a diverse representation of plant diseases. 10 plant diseases from the *PlantVillage* dataset are selected, including *Apple black rot*, *Apple healthy*, *Apple rust*, *Apple scab*, *Blueberry healthy*, *Cherry healthy*, *Cherry powdery mildew*, *Corn common rust*, *Corn gray leaf spot*, and *Corn healthy*. The details of dataset for transfer learning are shown in **Table 2**. In our experiment, each category of the disease dataset was randomly divided into a training set and a test set at the rate of 7:3. The training set contained 8143 images, the test set contained 3867 images, and the whole dataset contained 12010 images in total.

**Table 2:** *PlantVillage* transfer learning dataset.

ID	Name	Training set size	Test set size
1	*Apple black rot*	700	300
2	*Apple scab*	700	300
3	*Apple healthy*	1151	493
4	*Apple rust*	700	300
5	*Blueberry healthy*	1051	451
6	*Cherry healthy*	739	317
7	*Cherry powdery mildew*	700	300
8	*Corn common rust*	836	356
9	*Corn gray leaf spot*	700	300
10	*Corn healthy*	816	350

**Figure 2:**
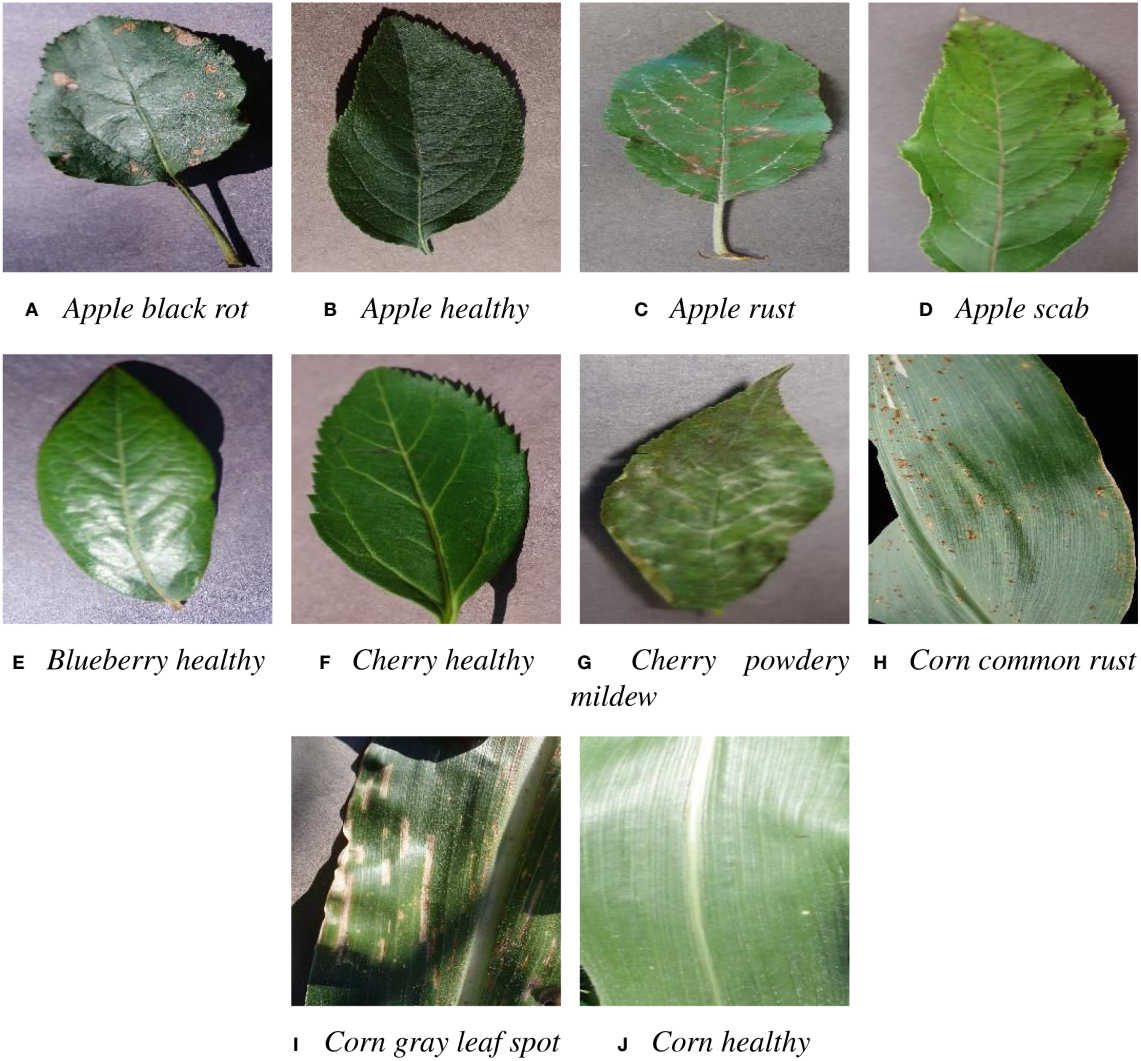
Examples of *PlantVillage* dataset for transfer learning.

By including a wide range of plant diseases, the effectiveness of the proposed transfer learning method in addressing the challenge of limited training data for specific plant diseases can be evaluated. Furthermore, this diverse dataset can be used for the developing a more robust and generalized model, which can be applied to a wider range of plant disease diagnosis and monitoring tasks. **Figure 2** is an illustration of 10 plant disease images in the *PlantVillage* dataset for transfer learning in our experiments.

## 3 Methods

### 3.1 Data preprocessing

To ensure uniformity across the dataset and elevate the quality of input data, we adhered to the following pivotal procedures:

1. **Image format standardization:** To maintain computational consistency, all images were transmuted into a 3-channel RGB configuration.
2. **Dimensional homogenization:** Conforming to the prerequisites of the model input, every image was adjusted to a resolution of 64 ∗ 64 pixels.
3. **Random cropping strategy:** Aiming to enhance the model’s focal attributes, random cropping was employed with the image’s centroid serving as the pivot.
4. **Image normalization techniques:** Every image underwent a meticulous normalization procedure, with pixel values scaled between 0 and 1, complemented by a subtle Gaussian blur to attenuate potential noise disturbances.

### 3.2 SENet network

In this paper, the SENet neural network was used, which is an improved version of the ResNet50 architecture (Joshy and Rajan, 2023; Zhu et al., 2023). The ResNet50 neural network is characterized by consisting of an initial convolutional layer, four Layer layers, two pooling layers, and one softmax activation layer. Each Layer layer is composed of multiple residual blocks, enhancing the network’s ability to effectively propagate gradients and learn complex features.

SENet incorporates a Squeeze-and-Excitation (SE) module after each residual block of ResNet50, which enhances the network’s capacity for selective feature emphasis. This module is designed to selectively weight the feature maps produced by the previous layer based on their importance for classification. Therefore, SENet allows for more focused attention on pertinent features in the input image, significantly improving performance in fine-grained image recognition tasks.

The choice of SENet for rice disease phenotype recognition is motivated by its advanced feature recalibration capability, which significantly enhances the model’s focus on relevant features. SENet’s unique Squeeze-and-Excitation block dynamically adjusts channel-wise feature responses, allowing the model to emphasize informative features while suppressing less useful ones. This is particularly effective in rice disease recognition, where distinguishing between subtle variations in disease symptoms is crucial. SENet’s ability to adaptively recalibrate feature maps leads to more accurate and robust recognition of rice disease patterns, making it an ideal choice for this application.

**Table 3** provides a detailed summary of the ResNet50 architecture used in this paper, including the number of filters in each convolutional layer, the kernel size, and the stride. The final output of the ResNet50 network is input into the SENet module, culminating in a softmax probability distribution of the 10 plant subclasses in the *PlantVillage* dataset.

**Table 3:** Structure of ResNet50 used in this paper.

Layer name	Layer configuration
Initial convolution layer 3*3 max pool stride=2	7*7 64 stride=2
Layer1	$\left[\begin{array}{c} 1*1\;\;64\\ 3*3\;\;64\\ 1*1\;\;256 \end{array}\right]*3$
Layer2	$\left[\begin{array}{c} 1*1\;\;128\\ 3*3\;\;128\\ 1*1\;\;512 \end{array}\right]*4$
Layer3	$\left[\begin{array}{c} 1*1\;\;256\\ 3*3\;\;256\\ 1*1\;\;1024 \end{array}\right]*6$
Layer4	$\left[\begin{array}{c} 1*1\;\;512\\ 3*3\;\;512\\ 1*1\;\;2049 \end{array}\right]*3$
Average Pool 1000-d fc softmax	

**Figure 3:**
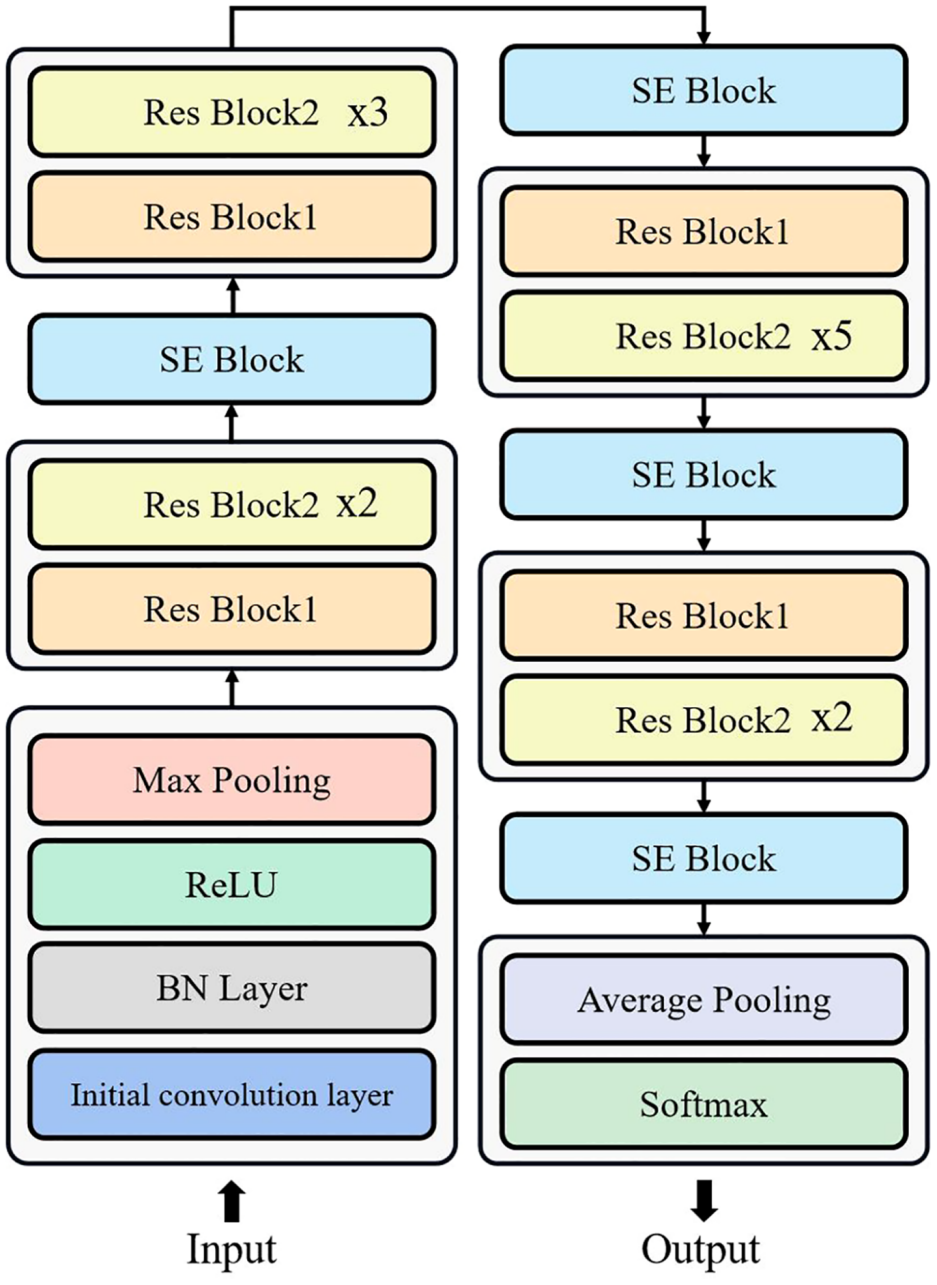
SENet network structure.

The structure of SENet is shown in **Figure 3**. In SENet, each layer comprises two distinct residual blocks. The first one is referred to as residual block one, which consists of three convolutional layers with a kernel size of 1*1, and an additional convolutional.

layer with a kernel size of 3*3. The input to residual block one is a matrix with a channel number of *C*, length of *L*, and width of *W*.

**Figure 4:**
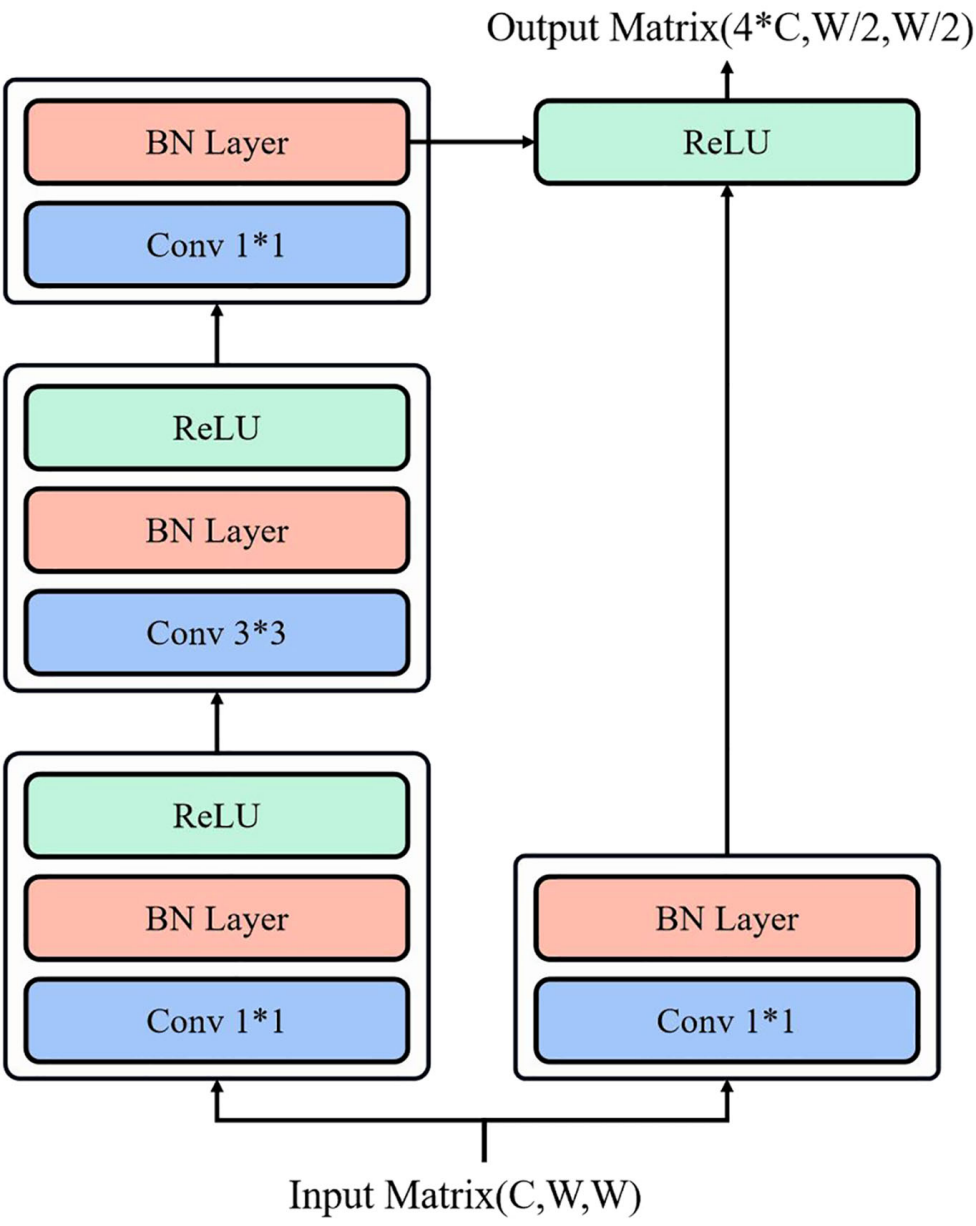
The first type of residual block structure.

However, in our work, we performed preprocessing on the images to resize both the length and width to *W*. Consequently, the input matrix dimensions of residual block one become (*C*,*W*,*W*). The output of residual block one is a matrix with 4C channels, and the length and width are halved, resulting in a data matrix of size (4*C*,*W*/2,*W*/2). Therefore, the residual block increases the number of channels to extract features. The first type of residual block structure is depicted in **Figure 4**.

**Figure 5:**
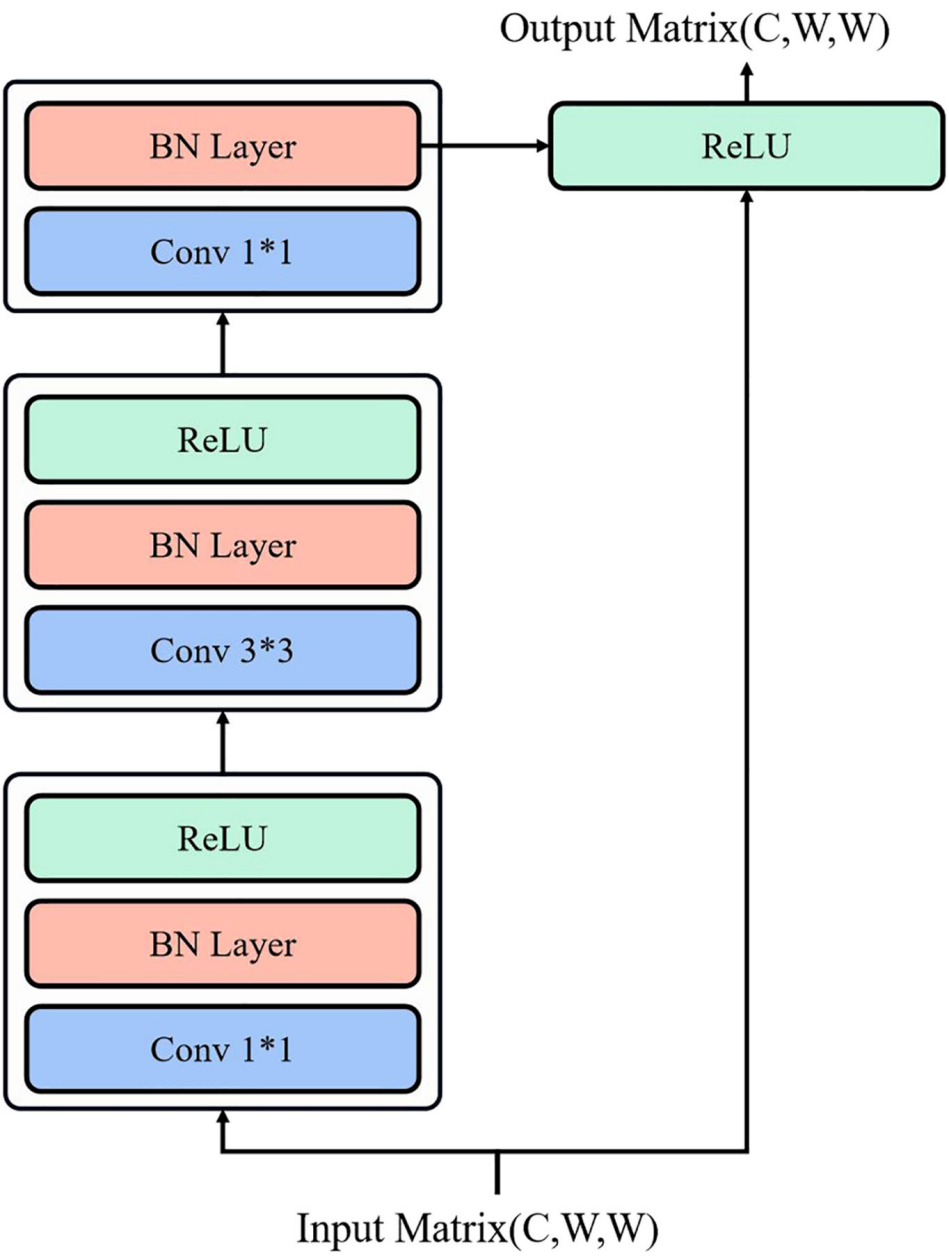
The second type of residual block structure.

The residual block 2 is used to accomplish identity mapping and illustrated in **Figure 5**. It is composed of two convolutional layers with a kernel size of 1*1, along with an additional convolutional layer with a kernel size of 3*3. Similar to residual block 1, the input to residual block 2 is a matrix with a channel number of *C*, length of *L*, and width of *W*. However, in our study, we performed preprocessing on the images to ensure that their length and width are both adjusted to *W*. Consequently, the input matrix dimensions of residual block 2 become (*C*,*W*,*W*). The output of residual block 2 remains identical to the input.

The number of convolution kernels in the convolution layer of each residual block in the model is summarized in **Table 4**.

**Table 4:** Output channels of each network layer.

Layer index	Network layer	Output channels
1	Input	0
2	conv	64
3	Maxpooling	64
4	conv	64
5	conv	64
6	conv	256
7	conv	256
8	conv	256
9	conv	256
10	conv	256
11	conv	256
12	conv	256
13	conv	128
14	conv	128
15	conv	512
16	conv	512
17	conv	512
18	conv	512
19	conv	512
20	conv	512
21	conv	512
22	conv	512
23	conv	512
24	conv	512
25	conv	256
26	conv	256
27	conv	1024
28	conv	1024
29	conv	1024
30	conv	1024
31	conv	1024
32	conv	1024
33	conv	1024
34	conv	1024
35	conv	1024
36	conv	1024
37	conv	1024
38	conv	1024
39	conv	1024
40	conv	1024
41	conv	1024
42	conv	512
43	conv	512
44	conv	2048
45 46 47 48 49 50 51	conv conv conv conv conv conv Average pool	2048 2048 2048 2048 2048 2048 0

After four sets of layer, the final characteristic matrix is obtained. The last layer is the global average pooling layer, and the *softmax* is used as the activation function.

The *softmax* function is a commonly used probability distribution function in deep learning, which maps a set of output values **x** = [*x*_1_, *x*_2_,…, *x_n_*] to a probability distribution **p** = [*p*_1_, *p*_2_,…, *p_n_*]. The *softmax* function is given by **Equation 1**:


(1) $$\text{s}\text{o}\text{f}\text{t}\text{m}\text{a}\text{x}{\left(x\right)}_{i}\;=\frac{\text{e}\text{x}\text{p}\left(x_{i}\right)}{\sum_{j=1}^{n}\text{e}\text{x}\text{p}\left(x_{j}\right)},\;\text{f}\text{o}\text{r}\;i\;=\;1,\;2,\;\ldots ,\;n$$

The *softmax* function exponentiates each element of the input vector **x** and then normalizes them to ensure that their sum equals to 1. The resulting values **p** can be interpreted as class probabilities in multi-class classification tasks. The *softmax* function simplifies the computation of loss functions and the optimization of models.

### 3.3 Transfer learning of TRiP

Transfer learning aims to leverage knowledge acquired from previously trained tasks to solve related tasks more efficiently (Abbas et al., 2021; Rajpoot et al., 2023). In the context of rice disease recognition, this approach is particularly valuable due to the limited availability of large, annotated datasets specific to this task. This approach mitigates issues associated with overfitting when training on small datasets and reduces the time needed for training on large datasets (Fan et al., 2022; Saberi Anari, 2022).

**Figure 6:**
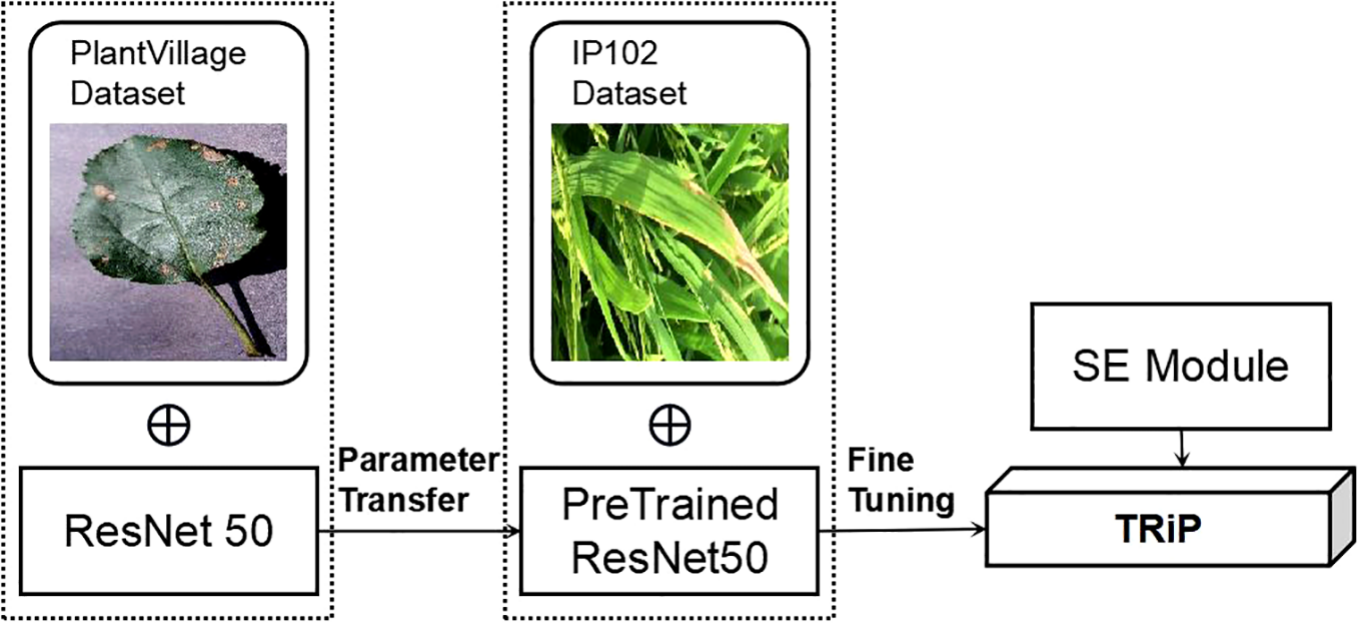
The framework of transfer learning of *TRiP*.

As illustrated in **Figure 6**, we first train the SENet on the *PlantVillage* dataset to obtain a pre-trained model. This pre-training step enables the SENet to learn general features of plant diseases, which can then be fine-tuned for the specific task of rice disease recognition. Then, we incorporate the parameters of the pre-trained model as transfer knowledge into the network training of the small dataset for the target task. This fine-tuning process tailors the model to the specific characteristics of rice diseases, enhancing its accuracy and efficiency for this application. Finally, we obtain a new model tailored to solving the target task. The integration of transfer learning with SENet thus allows for effective utilization of existing knowledge, significantly improving the performance of rice disease recognition.

By combining transfer learning with the SENet architecture, we effectively leveraged prior knowledge to enhance rice disease recognition. To fine-tune the pre-trained SENet model for the new task, we added new layers to the network and retrained the model on a small dataset of rice disease images.

The heuristic principle is adopted to determine which layers were most suitable for fine-tuning. Generally, the early layers of a network capture more generic features, such as edges and textures, while the later layers capture task-specific features. Thus, we typically fine-tune the later layers while keeping the early layers fixed. This process, known as fine-tuning, involves adjusting the parameters of the pre-trained model to the new task, enhancing its ability to handle task-specific features and improve performance on the rice disease recognition task.

In this paper, we utilized the cross-validation skill to determine the optimal fine-tuning strategy to enhance the performance of our SENet model in the task of rice disease recognition. Initially, we designed a candidate set encompassing a variety of potential fine-tuning strategies. These strategies considered the unfreezing of different numbers of layers, such as the last layer, the last two layers, the last three layers, and so on.

### 3.4 Attention mechanism

The attention mechanism has emerged as a pivotal technique in fine-grained image recognition, enhancing the nuances and subtleties of images (Lee et al., 2022; Hu et al., 2023; Jiang et al., 2023). The attention mechanism rapidly scans the global context, pinpoints the pertinent target regions, and dampens unrelated information. Consequently, the significant areas without necessitating auxiliary labeling information can be emphasized (Liu et al., 2022; Peng et al., 2023). Mathematically, the attention mechanism can be represented as **Equation 2**:


(2) $$\text{A}\text{t}\text{t}\text{e}\text{n}\text{t}\text{i}\text{o}\text{n}\left(Q,\;K,\;V\right)\;=\;\text{s}\text{o}\text{f}\text{t}\text{m}\text{a}\text{x}\left(\frac{QK^{T}}{\sqrt{d_{k}}}\right)V$$

In Equation 2, *Q* is the query vector and *K* and *V* stand for matrices of key and value vectors, respectively. *d_k_* denotes the dimensionality of the keys. The attention process can be broken down into three core steps:

Gauging the similarity between the query and each key. Normalizing these similarity scores via a softmax function to derive attention weights. Taking a weighted sum of the value vectors, modulated by the aforementioned attention weights. This attention mechanism’s dynamism and adaptability lead to a marked enhancement in performance compared to conventional models. Its strength lies in its capacity to zoom into specific input components based on their relevance to the current query (Tang et al., 2023).

There are two broad types of attention mechanisms: self-attention and soft attention (Yang et al., 2023). This paper makes use of the advantage the of the soft attention mechanism with the Squeeze-and-Excitation Networks (SENet). Within our research, the soft attention mechanism hones in on regional aspects, assigning them attention weights between 0 and 1. This determinate attention profile is crafted through rigorous training, empowering the network to fine-tune feature selection based on these trained weights.

### 3.5 Cross-validation

In this paper, the 10-fold cross-validation is adopted for testing the results. The dataset was divided into 10 subsets. For each iteration, one subset was used for validation while the other nine for training, cycling through all subsets. This approach reduces data-specific biases, ensuring a thorough evaluation. Importantly, we used precision as our evaluation metric, prioritizing the accuracy of positive identifications in our rice disease phenotype detection task.

## 4 Experimental results and analysis

### 4.1 Experimental environment

The experiments were conducted on CentOS 2.1903 64-bit, included 8 vCPU cores and an NVIDIA V100 GPU with 32 GB of memory. The algorithms were implemented with Tensorflow 1.14.0, Keras 2.25, Python 3.6. GPU and CUDA 11.8.

### 4.2 Experimental parameters

The size of image pixel segmentation *S* is 64*64, the number of input channels *C* is RGB-3 color, the batch size of batch *B* is 8, the number of iterations *E* is 50, the discard rate *P* is 0.5, momentum *β* is 0.9, the initial learning rate η is 1*e* ^−2^, and the fixed learning rate attenuates to 1*e* ^−2^. We use VGG16, ResNet50, Inception as the baseline for comparison.

### 4.3 Results evaluation metrics

We evaluate the results using accuracy, Macro-Precision, Macro-recall, Macro-*F*_1_, and Cross entropy loss. In the following definition, *TP* and *TN* refer to correct predictions of positive and negative examples, respectively. *FP* and *FN* represent incorrect predictions, where *FP* is a positive example incorrectly classified as unfavorable, and *FN* is a negative example incorrectly classified as positive.

1. **Accuracy** (*A*) primarily measures the overall effectiveness of the model and indicates the proportion of correctly predicted samples among all samples. The equation is shown as **Equation 3**:


(3) $$A=\frac{\text{T}\text{P}\;+\;\text{T}\text{N}}{\text{T}\text{P}\;+\;\text{T}\text{N}\;+\;\text{F}\text{P}\;+\;\text{F}\text{N}}$$

2. **Macro-Precision** (*P*_macro_) is chiefly concerned with the prediction results, specifically the average precision across all classes. It represents the proportion of samples that are correctly identified as positive, and is illustrated as **Equation 4**:


(4) $$P_{\text{m}\text{a}\text{c}\text{r}\text{o}}=\frac{1}{n}\sum_{i=1}^{n}\frac{\text{T}{\text{P}}_{i}}{\text{T}{\text{P}}_{i}+\;\text{F}{\text{P}}_{i}}$$

3. **Macro-Recall** (*R*_macro_) focuses on the true positive rate among actual positive samples. It calculates the average recall across all classes, and is given by **Equation 5**:


(5) $$R_{\text{m}\text{a}\text{c}\text{r}\text{o}}=\frac{1}{n}\sum_{i=1}^{n}\frac{\text{T}{\text{P}}_{i}}{\text{T}{\text{P}}_{i}+\;\text{F}{\text{N}}_{i}}$$

4. **Macro- F_1_ -Score** ( $F_{1_{macro}}$ ) is the harmonic mean between Macro-Precision and Macro-Recall, offering a balanced measure that takes both into account. The equation is defined as **Equation 6**:


(6) $$F_{1_{\text{m}\text{a}\text{c}\text{r}\text{o}}}=2\times \frac{P_{\text{m}\text{a}\text{c}\text{r}\text{o}}\times R_{\text{m}\text{a}\text{c}\text{r}\text{o}}}{P_{\text{m}\text{a}\text{c}\text{r}\text{o}}+R_{\text{m}\text{a}\text{c}\text{r}\text{o}}}$$

5. **Cross Entropy Loss** (*L*_ce_) It is commonly used for classification and identification tasks. The cross-entropy function for multi-classification problems is shown in the formula, where **y** is the accurate probability distribution, **q** is the predicted probability distribution, **N** is the number of samples, and **K** is the number of label values. The equation is shown as **Equation 7**:


(7) $$L_{ce}\left(y,\;q\right)\;=\;-\frac{1}{N}\sum_{i=1}^{K}\sum_{j=1}^{K}y_{i\;j}\;\text{l}\text{o}\text{g}\left(q_{i\;j}\right)$$

### 4.4 Non-transfer learning experimental results

#### 4.4.1 Training loss result

In this paper, the performance of four different types of neural networks, i.e. ResNet50, VGG16, Inception, and the method proposed in this paper, was evaluated using non-transfer learning. The non-transfer learning method involves training the network from scratch on the given dataset, without leveraging any pre-existing knowledge from other datasets.

**Figure 7:**
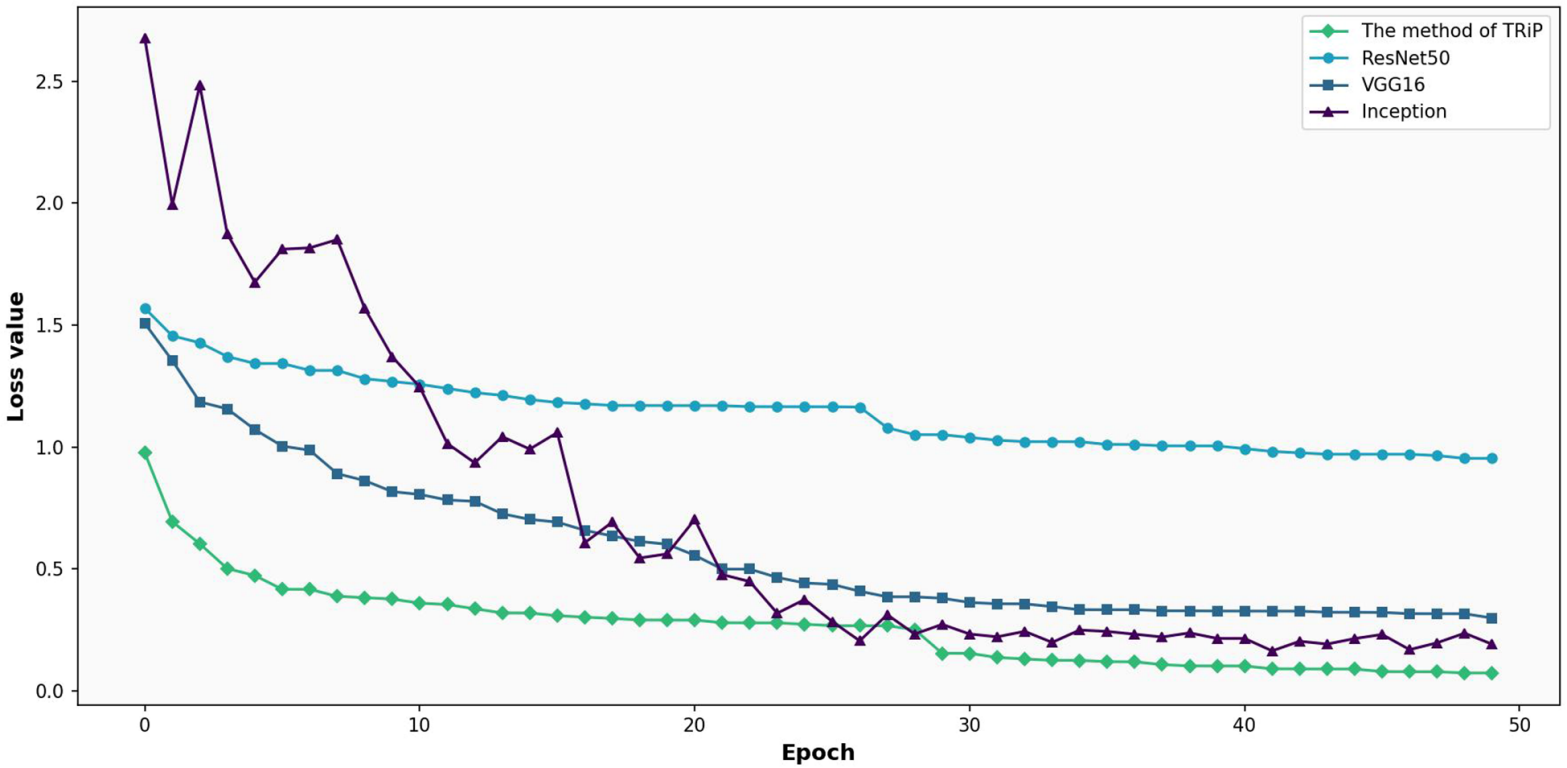
Comparative analysis of loss functions for four networks under non-transfer learning on the training set.

**Figure 7** gives the comparative analysis of loss functions for four networks under nontransfer learning on the training set. In **Figure 7**, a comparison of loss functions for four distinct networks Inception, ResNet50, VGG16, and the avant-garde *TRiP* is provided, all within the realm of non-transfer learning on a training set. As the iterations progress, all networks exhibit a downward trajectory in loss values, indicating ongoing learning refinement. Of particular note is the *TRiP*, which starts with an impressive low of 0.66 and swiftly converges to near-zero values in approximately 30 iterations, marking its pinnacle of adaptability and efficiency. Inception, in contrast, commences with a significant loss of 1.90 and diminishes at a more measured pace, suggesting possible adaptability nuances. ResNet50’s trajectory, starting at 1.49 and seeing a steady decline, underscores its unwavering stability, while VGG16, beginning at 1.34, displays competence but might benefit from extended iterations for optimum convergence.

**Figure 8:**
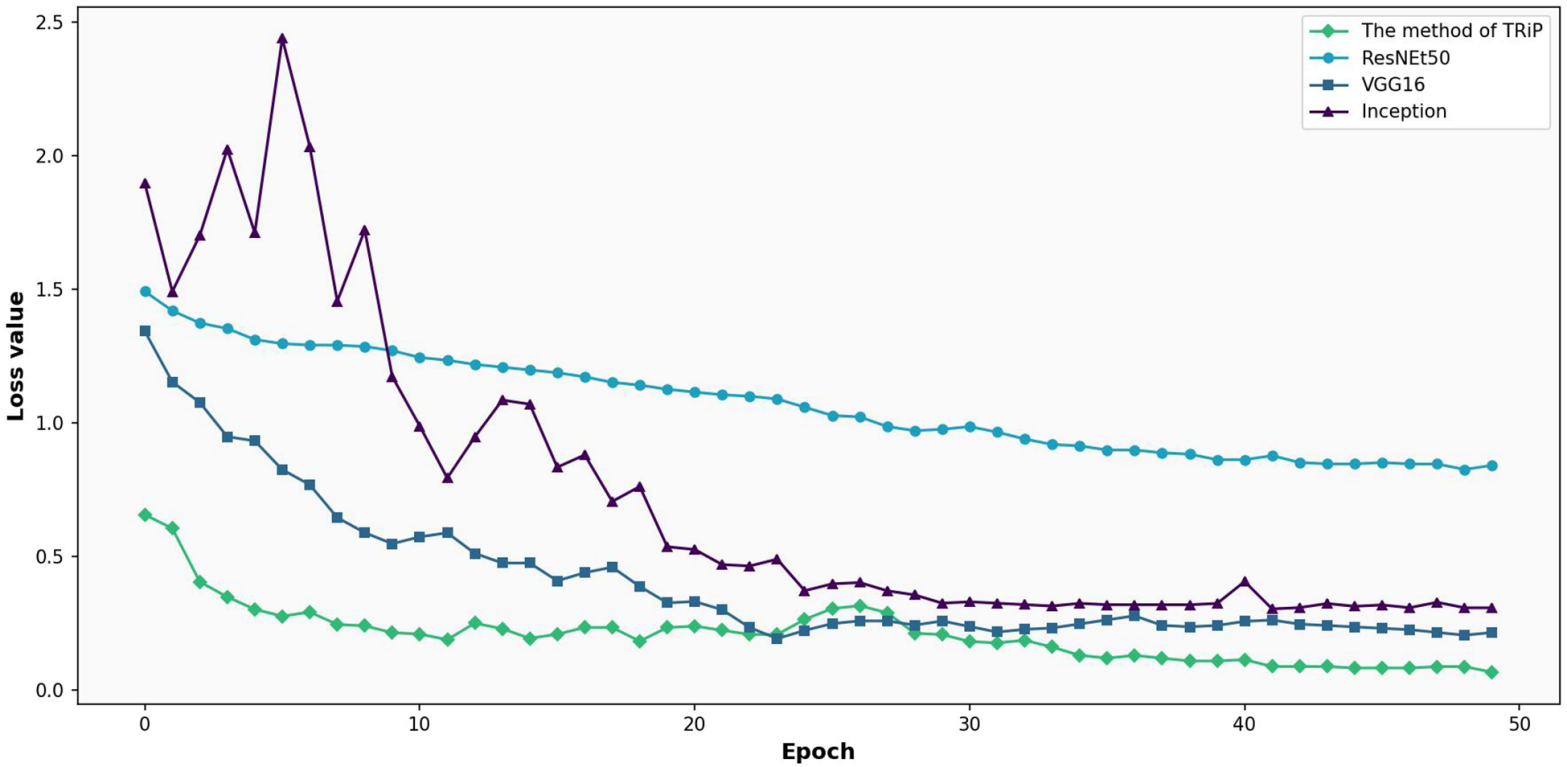
Comparative analysis of loss functions for three networks under non-transfer learning on the test set.

**Figure 8** is the comparative analysis of loss functions for three networks under non-transfer learning on the test set. Right from the outset, Inception’s fluctuating journey, commencing with a loss of 1.90 and seeing erratic oscillations, signals challenges in dataset generalization. Contrarily, both ResNet50 and VGG16 begin with losses of 1.49 and 1.34 respectively and showcase a consistent decline, championing their adaptability. However, deeper into the iterations, ResNet50 and VGG16 maintain a tempered reduction, with ResNet’s lowest reaching 0.8463 and VGG16’s touching 0.2051. Meanwhile, Inception’s remains tumultuous.

#### 4.4.2 Accuracy result

**Table 5** presents the accuracy of different network training methodologies rooted in non-transfer learning, it is evident that the *TRiP* method stands superior, achieving the highest accuracy of 0.9313. VGG16 closely follows with an accuracy that is 1.33% lower, while Inception lags by 2.99%. Of note, ResNet50, despite its wide acclaim in numerous applications, registered the lowest accuracy in this context, being 5.27% less accurate than the *TRiP* method. This indicates that, for the dataset and conditions under review, *TRiP* offers the most promising results, with VGG16 and Inception as worthy contenders, whereas ResNet50 may not be the optimal choice.

**Table 5:** Accuracy of network training set based on non-transfer learning.

Method	Accuracy	Relative change
The method of *TRiP*	0.9313	–
ResNet50	0.8822	5.27%
VGG16	0.9189	1.33%
Inception	0.9034	2.99%

**Table 6** delineates the accuracy metrics for various network test methodologies predicated upon non-transfer learning. The *TRiP* method emerges as the most effective, registering an accuracy of 0.9417. In its wake, VGG16 records an accuracy rate of 0.9213, trailing *TRiP* by 2.17%. Inception is only marginally behind VGG16 with an accuracy of 0.9179, indicating a 2.53% decrease compared to *TRiP*. It’s significant to highlight that ResNet50, in this analysis, lags notably with an accuracy score of 0.8379, showing a reduction of 4.68% in comparison to the *TRiP* method. The result means that for the specific test set and context, *TRiP* maintains its lead in accuracy, with VGG16 and Inception closely contesting, while ResNet50 is discernibly less effective.

**Table 6:** Accuracy of network test set based on non-transfer learning.

Method	Accuracy	Relative change
The method of *TRiP*	0.9417	–
ResNet50	0.8379	4.68%
VGG16	0.9213	2.17%
Inception	0.9179	2.53%

Based on the above experimental results, the performance of the method in this paper is the best under non-transfer learning because this network model introduces an SE module and attention mechanism, which can bias some specific features of the input image, and has a better effect on identifying fine-grained images such as rice disease images.

### 4.5 Transfer learning experimental results

#### 4.5.1 Training loss result

**Figure 9:**
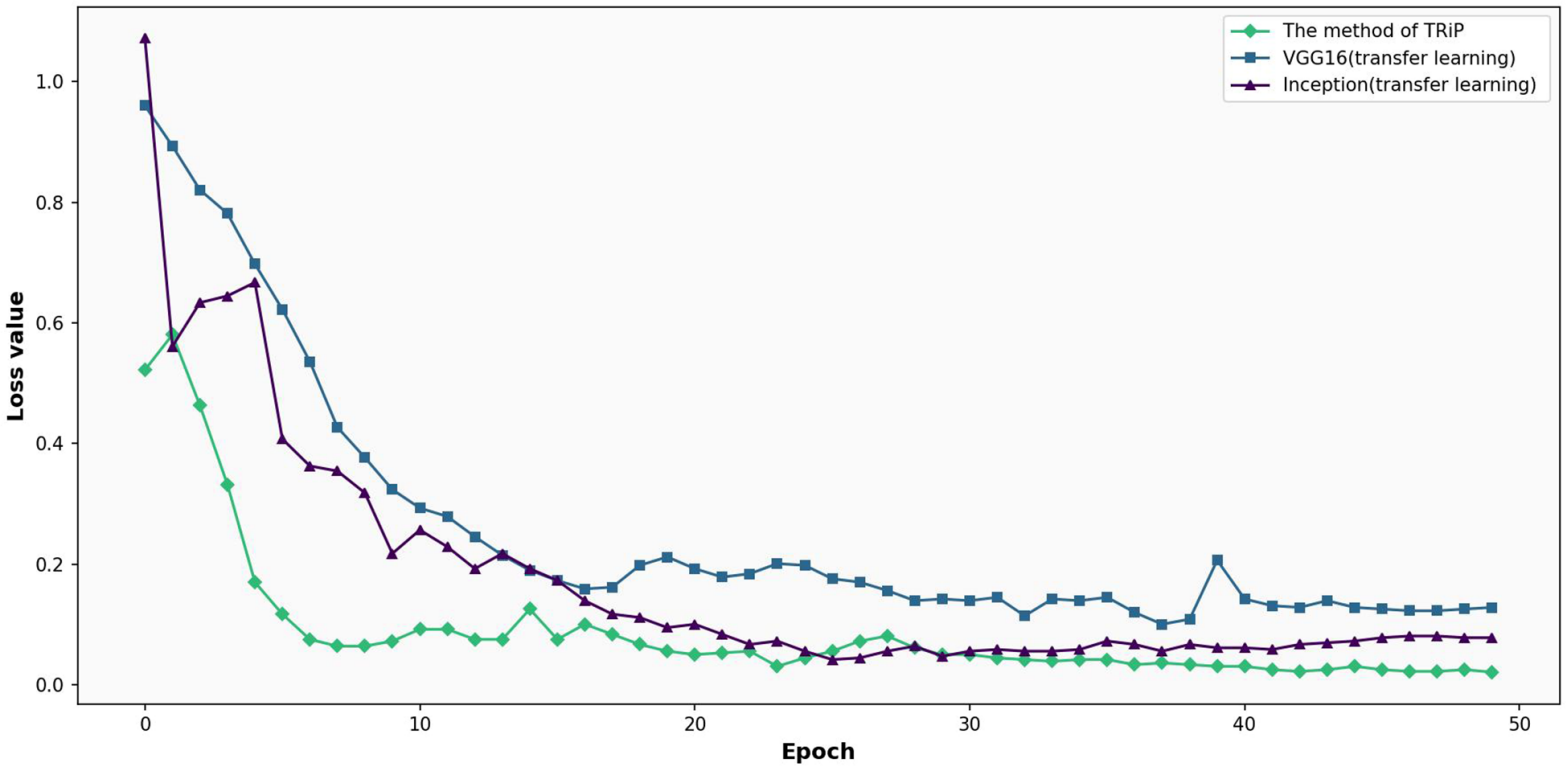
Comparative analysis of loss functions for three networks under transfer learning on training set.

The results of transfer learning method are compared with the three kinds of networks, VGG16 and Inception on the training set and test set. **Figure 9** shows the comparative analysis of loss functions for three networks under transfer learning on training set. In **Figure 9**, under the transfer learning framework on a training set, the loss functions of Inception, VGG16, and the *TRiP* method are compared. The *TRiP* method starts with a distinct advantage, registering an initial loss of 0.5219, in contrast to Inception’s 1.0716 and VGG16’s 0.9600. Through subsequent iterations, *TRiP* consistently manifests superior convergence, reaching a loss of 0.1702 at the 5th iteration. The loss of Inception and VGG16 methods reached 0.6670 and 0.6977, respectively. At the 19th iteration, while VGG16 approaches a loss of 0.1981 and Inception achieves 0.1116, *TRiP*’s loss changed slightly.

**Figure 10:**
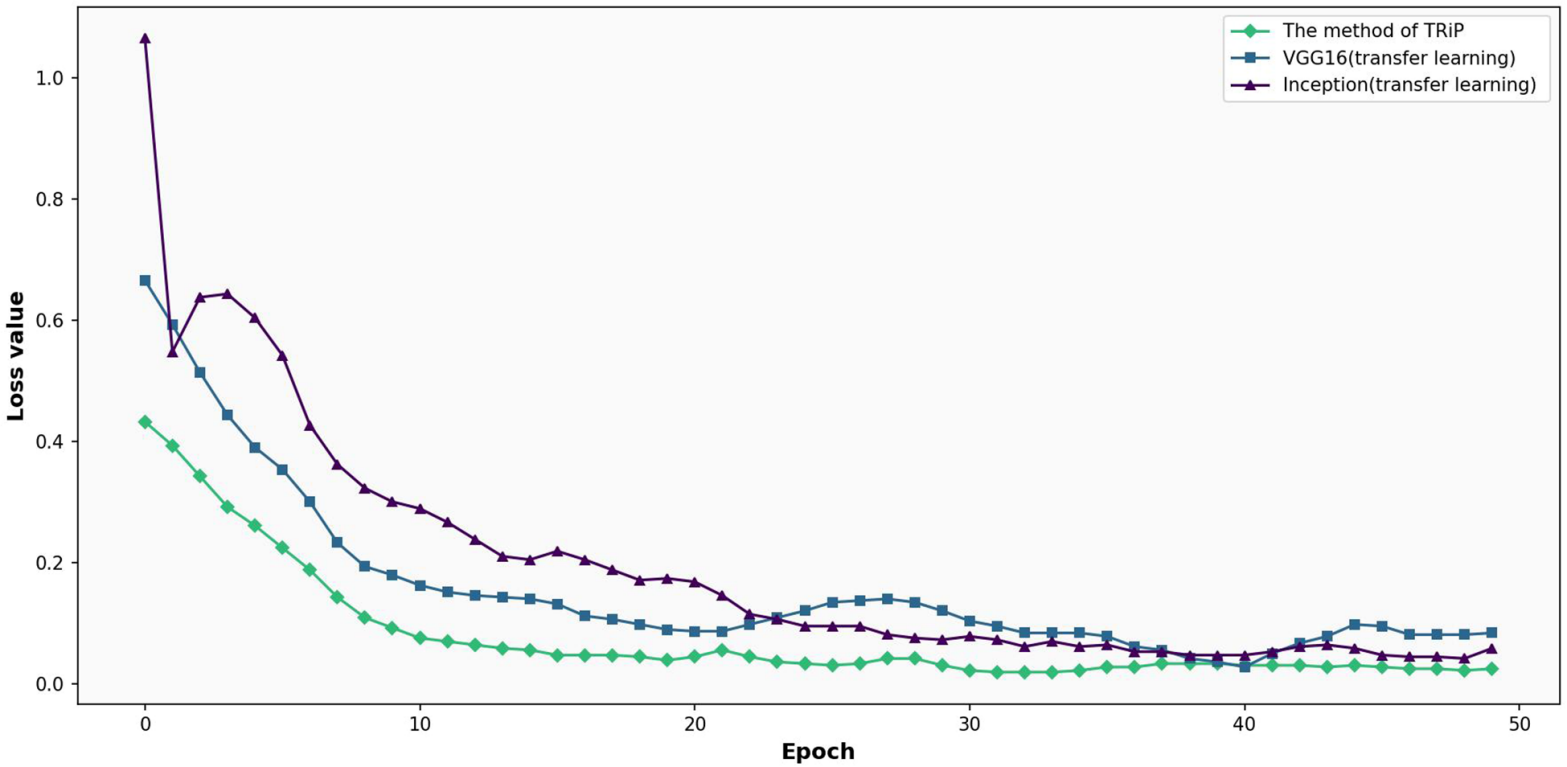
Comparative analysis of loss functions for three networks under transfer learning on test set.

**Figure 10** presents the comparative analysis of loss functions for three networks under transfer learning on test set. In **Figure 10**, focusing on a test set, the comparative analysis of the three networks unfolds. *TRiP* immediately stands out with an initial loss of 0.4328, a significant improvement over Inception’s 1.0651 and VGG16’s 0.6660. Progressing through the iterations, *TRiP*’s advantage becomes even more pronounced, recording a loss of 0.2614 by the 5th iteration, overshadowing Inception’s 0.6042 and VGG16’s 0.3906. By the 20th iteration, *TRiP*’s loss is approximately 0.0393. In comparison, Inception and VGG16 stabilize at 0.1742 and 0.0899, respectively. Across the evaluation, *TRiP* consistently demonstrates optimal performance in terms of convergence, outclassing both Inception and VGG16.

#### 4.5.2 Accuracy result

**Table 7** gives the accuracy of different network on the training set when transfer learning is applied. The *TRiP* method consistently demonstrates robust results, achieving a top accuracy of 0.9521. When transfer learning is employed with VGG16, its accuracy noticeably improves, reaching 0.9363, which is still 1.66% less than that achieved by *TRiP*. The accuracy of Inception when transfer learning is 0.9282, a 2.51% reduction compared to *TRiP*. The data emphasizes the efficacy of transfer learning in enhancing model performance, albeit *TRiP* retains its predominant position in this comparative evaluation.

**Table 7:** Accuracy of network training set based on transfer learning.

Method	Accuracy	Relative change
The method of *TRiP*	0.9521	–
VGG16(transfer learning)	0.9363	1.66%
Inception(transfer learning)	0.9282	2.51%

**Table 8** shows the accuracy utilizing transfer learning on the test set. From **Table 8**, the *TRiP* method once again excels, achieving an unparalleled accuracy of 0.9573. When VGG16 benefits from transfer learning, its performance escalates, reaching an accuracy of 0.9423, yet it remains 1.57% shy of the *TRiP* method. Similarly, leveraging transfer learning with Inception results in an accuracy score of 0.9332, which is 2.52% less compared to *TRiP*. These figures accentuate the potent impact of transfer learning in enhancing model precision. Nonetheless, the *TRiP* method remains the benchmark, delivering superior outcomes even in the face of enhanced competitors.

**Table 8:** Accuracy of network test set based on transfer learning.

Method	Accuracy	Relative change
The method of *TRiP*	0.9573	–
VGG16(transfer learning)	0.9423	1.57%
Inception(transfer learning)	0.9332	2.52%

In conclusion, the proposed method performs the best when transfer learning is used, due to the reason that transfer learning allowing for pre-training on a larger dataset, which provides more generalization ability compared to direct training on a small dataset used in non-transfer learning.

### 4.6 Results analysis

**Table 9** shows the results of the Macro-precision, Macro-recall rate, and Macro-*F*_1_ value, and comparisons with transfer learning and non-transfer learning on the test set. From **Table 9**, we can see that our method has the best performance; the Macro-precision, Macro-recall rate, and Macro-*F*_1_ are 0.9012 respectively. This is due to the introduction of the SE module with an increased attention mechanism to enhance the recognition ability of the model for fine-grained rice disease images and the use of transfer learning methods to reduce the over-fitting of the model due to the need for more sample data.

**Table 9:** Macro-level precision, recall, and *F*_1_ of different models.

Method	*P*_Macro_	*R*_Macro_	$F_{1_{Macro}}$
The method of *TRiP*	0.9012	0.9012	0.9012
SENet	0.8585	0.8585	0.8585
ResNet50	0.8434	0.8433	0.8434
VGG16	0.8571	0.8570	0.8571
VGG16 (Transfer Learning)	0.8793	0.8793	0.8793
Inception	0.8500	0.8500	0.8500
Inception (Transfer Learning)	0.8750	0.8750	0.8750

## 5 System implementation and deployment

### 5.1 Systems architecture of TRiP

The *TRiP* system is implemented based on PHP, MySQL and Redis, which can be used and deployed for edge computing. PHP-based RESTful APIs are adopted to regulate the client-server communications. The lightweight SQL server MariaDB is used for storing datasets of different formats, including images and phenotypic traits.

**Figure 11:**
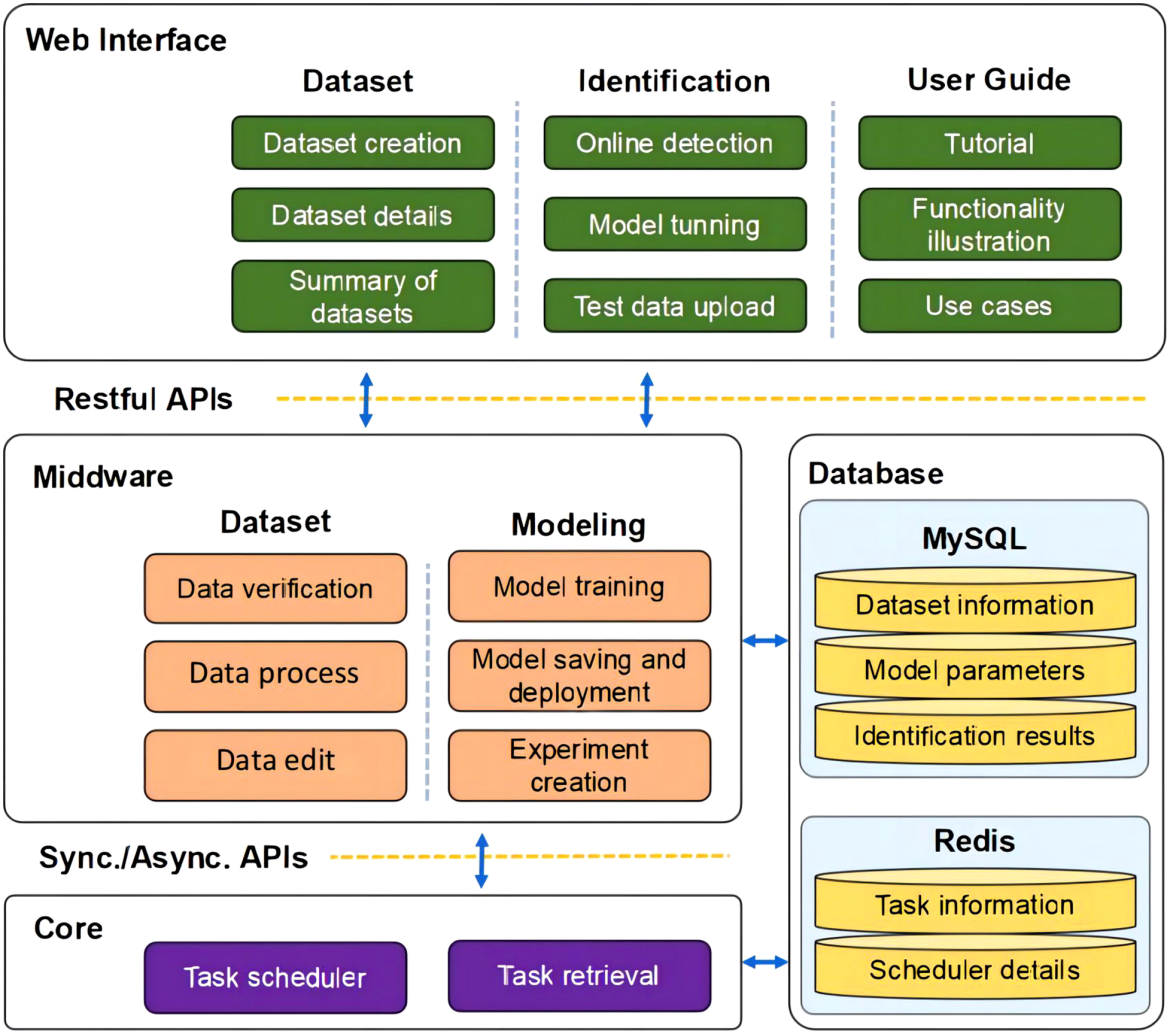
The *TRiP* system architecture of rice disease recognition.

As illustrated in **Figure 11**, the architecture of *TRiP* includes: 1)**Web interface**: Users can upload rice phenotype images through the web interface and view recognition results on the page; 2) **Middleware**: The middleware receives the uploaded images and passes them to the core module for recognition, and receives the model parameters provided by the user for model deployment; 3)**Core**: The core module uses a pre-trained SNet model for feature extraction and classification of the uploaded images. It can also use some pre-processing techniques to enhance the quality of the images. Task scheduling and task retrieval are performed; 4)**Database**: The database is responsible for storing the uploaded images and their corresponding recognition results. Keeps the relevant parameters of the model and the model performance.

### 5.2 The flowchart of TRiP

The basic idea of *TRiP* is Machine Learning as a Service (MLaaS), which utilizes machine learning algorithms and models as cloud services to tackle the challenging problem of rice disease classification. *TRiP* enables developers to build, deploy, manage machine learning models and performance monitoring based on the cloud infrastructure.

**Figure 12:**
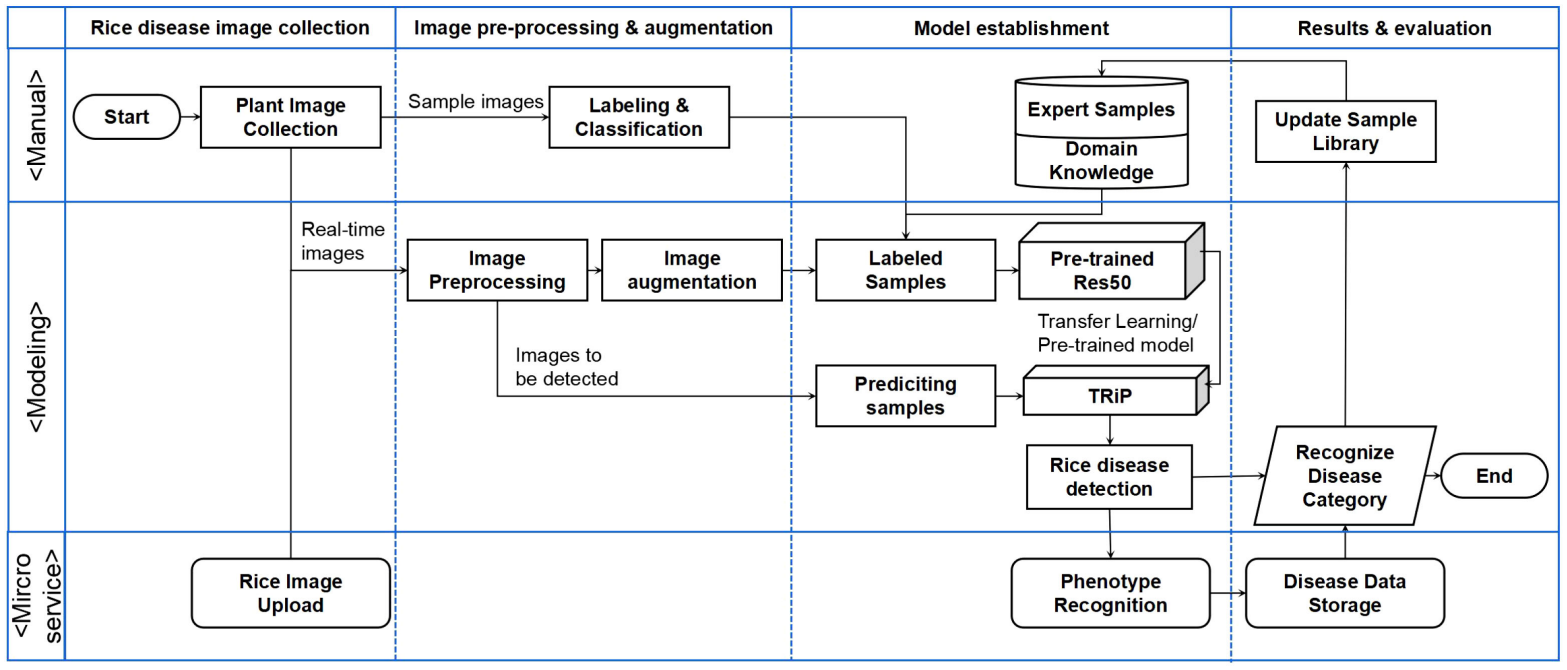
The flowchart of transfer learning-based rice disease phenotype recognition *TRiP* platform.

The system overview of *TRiP* is shown in **Figure 12**. The processing steps include: Firstly, the rice disease image samples are collected and annotated, with accurate annotations based on the knowledge of domain experts. Then, the obtained images are processed using image processing techniques, including grayscale conversion, image filtering, image sharpening, and resizing.

The data augmentation methods are used to increase the training dataset, such as random rotation, flipping, and translation. Subsequently, the sample images are input into the *TRiP* system for training. The model is pre-trained on the *PlantVillage* dataset firstly, then the transfer learning technique is employed and the model is migrated to the IP102 dataset for further optimization. After training and parameters fine-tuning, the model is used to predict the disease categories of rice disease.

### 5.3 Microservices of TRiP

**Figure 13:**
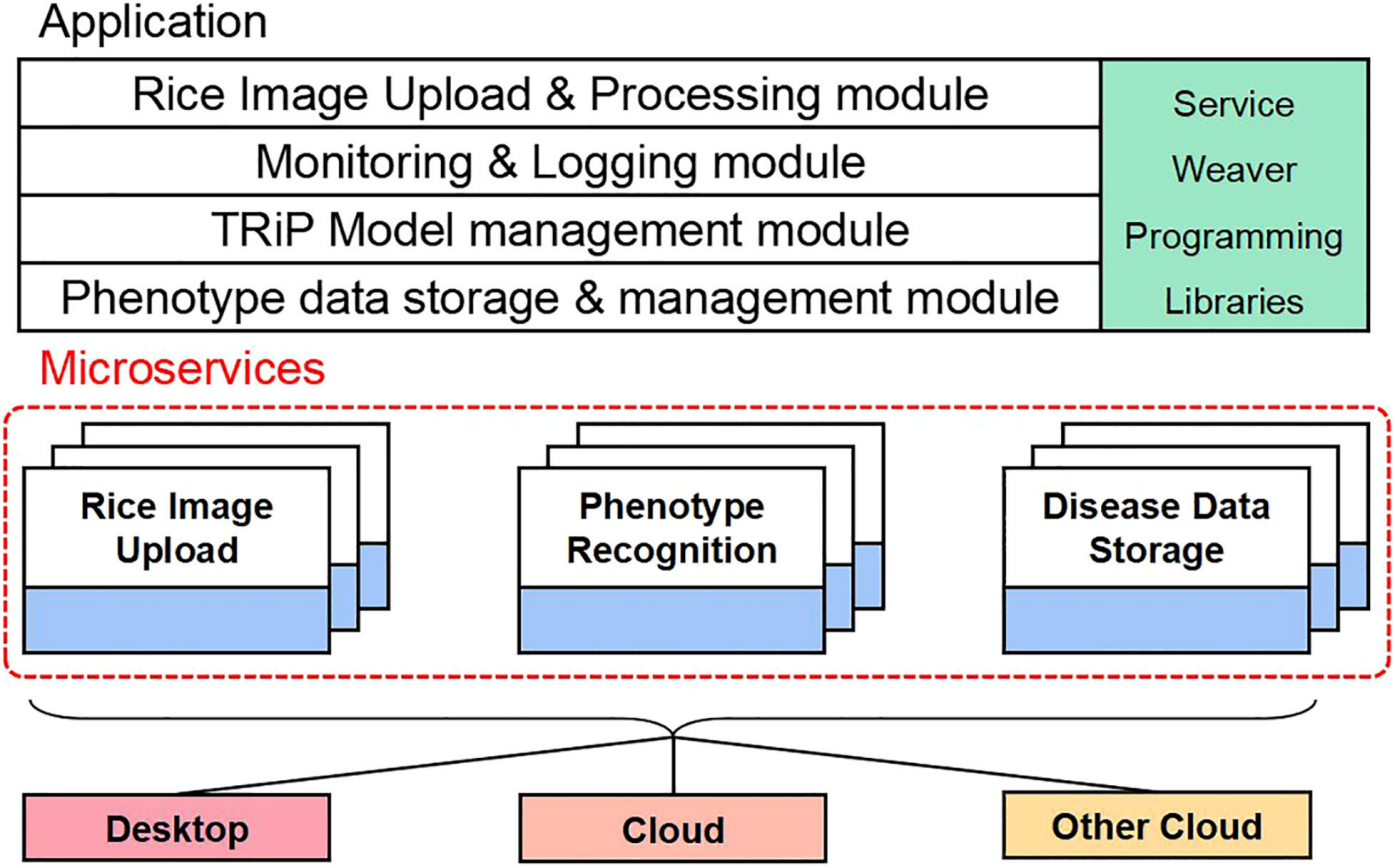
Microservice framework of *TRiP*.

The rice disease recognition system is developed using microservices architecture, such as Amazon Web Services (AWS). Microservices architecture is a loosely coupled architecture that splits a large application into a set of small and independent services. Each service can be developed, tested, deployed, and extended separately. The Microservice model structure of *TRiP* is shown in **Figure 13**.

### 5.4 The accessibility of TRiP

**Figure 14:**
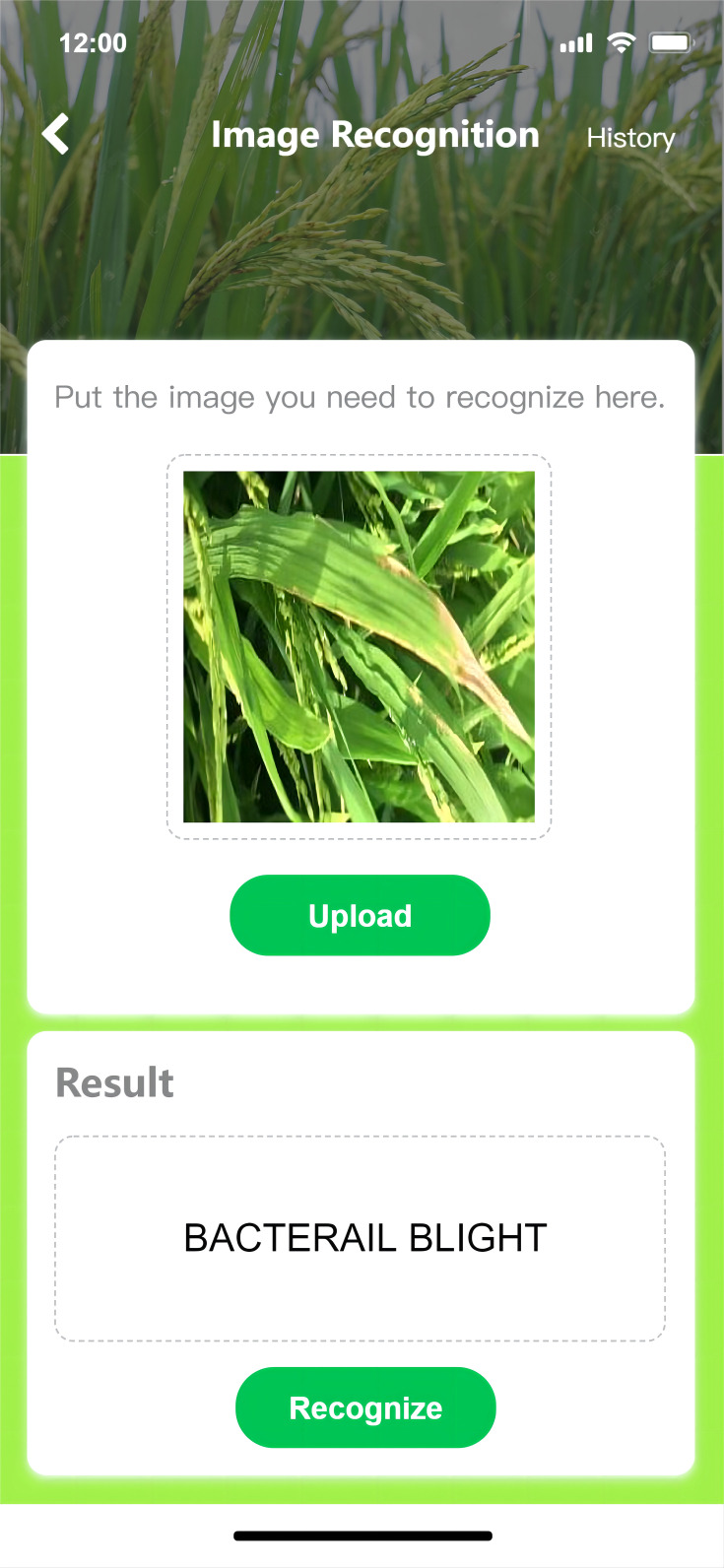
User interface of *TRiP*.

The user interface of *TRiP* is illustrated in **Figure 14**. The interface and interactive design of *TRiP* with navigation cues, succinct layouts, and pertinent prompts messages for easy uasge.

Furthermore, *TRiP*’s adaptability across multiple devices and can be used in the field or an office setting. This cross-device compatibility, refined for desktops, tablets, and mobile phones, guarantees a uniform experience for different users.

## 6 Conclusion

In this paper, we concentrated on identifying rice diseases such as *Bacterial blight*, *Blast*, *Brown spot*, *Leaf smut*, and *Tungro* by analyzing their phenotypes. We make use of the pre-trained *PlantVillage* database model parameters as the initial setting for our SENet network in a transfer learning context. We incorporated the Squeeze-and-Excitation (SE) module to enhance the feature extraction process, thus refining the network’s focus on critical disease indicators.

The performance of our trained SENet network was benchmarked against other established neural networks, demonstrating superior efficacy in disease identification. Additionally, we developed a rice disease detection platform employing a microservice architecture, tailored for efficient and scalable deployment in edge computing environments. Our research not only offers a promising method for accurate rice disease phenotyping but also provides a user-friendly and technologically advanced platform for agricultural disease recognition applications.

## Data availability statement

The raw data supporting the conclusions of this article will be made available by the authors, without undue reservation.

## Author contributions

PY: Data curation, Funding acquisition, Writing – review & editing, Project administration. YX: Data curation, Software, Writing – original draft. YT: Writing – review & editing. HX: Supervision, Writing – review & editing.
